# Supplementary material for: Antibacterial Activity of Amidodithiophosphonato Nickel(II) Complexes: An Experimental and Theoretical Approach
Source: Molecules. 2020 Apr 28;25(9):2052. doi: 10.3390/molecules25092052 (PMC7248947; doi:10.3390/molecules25092052)

Supplementary material

Antibacterial Activity of Amidodithiophosphonato Nickel (II) Complexes: An Experimental and Theoretical Approach

Enrico Podda [1], Massimiliano Arca [1], Giulia Atzeni [1], Simon J. Coles [2], Antonella Ibba [3], Francesco Isaia [1], Vito Lippolis [1], Germano Orrù [3,4], James B. Orton [2], Anna Pintus [1], Enrica Tuveri [3], M. Carla Aragoni [1,*]

[1] Dipartimento di Scienze Chimiche e Geologiche, Università degli Studi di Cagliari, Cittadella Universitaria, SS. 554 bivio Sestu, 09042 Monserrato – Cagliari, Italy.

[2] UK National Crystallography Service, School of Chemistry, Faculty of Engineering and Physical Sciences, University of Southampton, SO17 1BJ, United Kingdom.

[3] Department of Surgical Sciences, University of Cagliari, Cagliari, Italy.

[4] National Research Council of Italy, ISPA-CNR, Sassari, Italy

* aragoni@unica.it

Page 2 **Table S1:** Crystal data and refinement parameters for compounds $(BzNH_3)_2[Ni(dtp)_2]\cdot 2H_2O$, $(PhBuNH_3)_2[Ni(dtp)_2]$, and $(PhBuNH_3)_2[(ArPS_2)_2O]$.
Table S2: Hydrogen Bond information for $(BzNH_3)_2[Ni(dtp)_2]\cdot 2H_2O$.
Table S3: Hydrogen Bond information for $(PhBuNH_3)_2[Ni(dtp)_2]$.
Page 3 **Table S4**: Hydrogen Bond information for $(PhBuNH_3)_2[(ArPS_2)_2O]$.
Page 4 **Table S5:** Drug susceptibility profile of the bacterial strains used in this work.
Page 5 **Table S6** MIC, MBC, and MBIC values (μg/mL) towards bacterial strains determined for $NiCl_2\cdot 6H_2O$.
Table S7 MIC, MBC, and MBIC values (mol/L) towards bacterial strains determined for $NiCl_2\cdot 6H_2O$
Page 6 **Figure S1**: ^{1}H NMR spectrum of $(BzNH_3)$(BzNH-adtp) in DMSO-d_6 at 25 °C.
Figure S2: ^{1}H NMR spectrum of $(PhBuNH_3)$(PhBuNH-adtp) in DMSO-d_6 at 25 °C.
Page 7 **Figure S3**: ^{1}H NMR spectrum of $[Ni(BzNH\text{-}adtp)_2]$ in $CDCl_3$ at 25 °C.
Figure S4: ^{1}H NMR spectrum of $[Ni(PhBuNH\text{-}adtp)_2]$ in $CDCl_3$ at 25 °C.
Page 8 **Figure S5**: Packing view for $(BzNH_3)_2[Ni(dtp)_2]\cdot 2H_2O$ along the 010 direction.
Figure S6: Contents and numbering scheme of the asymmetric unit of $(PhBuNH_3)_2[Ni(dtp)_2]$ thermal ellipsoids drawn at the 50% probability level, disorder shown.
Page 9 **Figure S7:** Contents and numbering scheme of the asymmetric unit of $(PhBuNH_3)_2[(ArPS_2)_2O]$ thermal ellipsoids drawn at the 50% probability level, disorder shown.
Figure S8: Antimicrobial activity, as inhibition diameter ø of $[Ni(BzNH\text{-}adtp)_2]$ and $[Ni(PhBuNH\text{-}adtp)_2]$ against eight different microbial species, C = [200 μg/mL]. The bars represent the mean and lines indicate the standard error of the mean.
Page 10 **Figure S9**: ^{1}H NMR spectrum of $(BzNH_3)_2[Ni(dtp)_2]$ in DMSO-d_6 at 25 °C.
Figure S10: ^{1}H NMR spectrum of $(PhBuNH_3)_2[Ni(dtp)_2]$ in DMSO-d_6 at 25 °C.
Page 11 **Figure S11:** FT-IR spectrum of $(BzNH_3)$(BzNH-adtp).
Figure S12: FT-IR spectrum of $[Ni(BzNH\text{-}adtp)_2]$.
Figure S13: FT-IR spectrum of $(BzNH_3)_2[Ni(dtp)_2]$.
Page 12 **Figure S14:** FT-IR spectrum of $(PhBuNH_3)$(PhBuNH-adtp).
Figure S15: FT-IR spectrum of $[Ni(PhBuNH\text{-}adtp)_2]$.
Page 13 **Figure S16:** FT-IR spectrum of $(PhBuNH_3)_2[Ni(dtp)_2]$.
Figure S17: FT-IR spectrum of $(PhBuNH_3)_2[(ArPS_2)_2O]$.
Page 15 CheckCIF/PLATON report for $(PhBuNH_3)_2[(ArPS_2)_2O]$.
Page 20 CheckCIF/PLATON report for $(BzNH_3)_2[Ni(dtp)_2]\cdot 2H_2O$.
Page 23 CheckCIF/PLATON report for $(PhBuNH_3)_2[Ni(dtp)_2]$.

Table S1: Crystal data and refinement parameters for compounds $(BzNH_3)_2[Ni(dtp)_2]\cdot 2H_2O$, $(PhBuNH_3)_2[Ni(dtp)_2]$, and $(PhBuNH_3)_2[(ArPS_2)_2O]$.

	$(BzNH_3)_2[Ni(dtp)_2]\cdot 2H_2O$	$(PhBuNH_3)_2[Ni(dtp)_2]$	$(PhBuNH_3)_2[(ArPS_2)_2O]$
Formula	$C_{28}H_{38}N_2NiO_6P_2S_4$	$C_{34}H_{46}N_2NiO_4P_2S_4$	$C_{78.5}H_{104}N_4O_6P_4S_8$
M	747.49	795.62	1580.01
Crystal System	triclinic	triclinic	triclinic
Space Group	P-1	P-1	P-1
a/ Å	6.8210(3)	15.6721(3)	14.0833(3)
b/ Å	8.4029(2)	16.7478(4)	15.6656(3)
c/ Å	15.5012(5)	18.1761(4)	20.0356(4)
α / °	97.111(2)	109.937(2)	96.779(2)
β / °	90.729(3)	106.923(2)	100.906(2)
γ / °	102.606(3)	106.836(2)	106.974(2)
V /Å^3	859.66(5)	3868.75(16)	4080.13(15)
Z	1	4	2
$\rho_{calcd.}$	1.444	1.366	1.286
T/K	100(2)	100(2)	100(2)
μ(Mo-Kα)/mm^{-1}	0.942	0.838	0.350
Collected Refl.	38286	44896	88236
Uniq. Refl.	3941	17089	18643
Refl. with I > 2σ(I)	3591	14095	13259
R_{int}	0.0356	0.0188	0.0338
$R1$ (I > 2σ(I))	0.0258	0.0395	0.0583
$wR2$ (all data)	0.0666	0.1112	0.1540

Table S2: Hydrogen Bond information for $(BzNH_3)_2[Ni(dtp)_2]\cdot 2H_2O$.

D	H	A	d(D-H)/Å	d(H···A)/Å	d(D···A)/Å	D-H···A/deg
N11	H11C	O1	0.92(2)	1.84(2)	2.7412(17)	166.2(17)
N11	H11E	O21'	0.87(2)	1.91(2)	2.745(5)	161.7(18)
N11	H11E	O21A'	0.87(2)	2.01(2)	2.806(11)	152.4(17)
O21	H21B	O1	0.87	1.82	2.692(5)	176.7
O21A	H21D	O1	0.87	1.83	2.705(11)	179.5

' = 1+x, y, z

Table S3: Hydrogen Bond information for $(PhBuNH_3)_2[Ni(dtp)_2]$.

D	H	A	d(D-H)/Å	d(H···A)/Å	d(D···A)/Å	D-H···A/deg
N41	H41D	O21'	0.91	1.81	2.701(2)	164.8
N41	H41E	O31	0.91	1.90	2.761(2)	156.6
N51	H51C	O21'	0.91	1.97	2.836(2)	159.7
N51	H51D	O1'	0.91	1.89	2.784(2)	165.2
N61	H61E	O31''	0.91	1.86	2.754(2)	165.4
N61	H61F	O11'''	0.91	1.84	2.722(2)	161.6
N71	H71K	O11	0.91	1.80	2.696(3)	166.8
N71	H71M	O1	0.91	1.84	2.734(3)	168.5

' = 1-x,1-y,1-z; '' = 1-x,2-y,1-z; ''' = x,1+y, z

Table S4: Hydrogen Bond information for $(PhBuNH_3)_2[(ArPS_2)_2O]$.

D	H	A	d(D-H)/Å	d(H···A)/Å	d(D···A)/Å	D-H···A/deg
N41	H41B	S22'	0.91	2.36	3.214(3)	157.0
N51	H51A	S24	0.91	2.29	3.113(4)	150.0
N51	H51B	S4	0.91	2.32	3.169(3)	154.0
N51	H51C	S21	0.91	2.34	3.211(3)	161.0
N71	H71A	S1	0.91	2.36	3.226(3)	160.0
N71	H71B	S2''	0.91	2.43	3.277(3)	155.0
N71	H71C	S4''	0.91	2.34	3.225(3)	164.0
C6	H6	O1	0.95	2.45	2.888(4)	108.0
C7	H7A	S22'	0.98	2.87	3.598(4)	132.0
C9	H9	O1	0.95	2.38	2.815(3)	108.0
C22	H22	O21	0.95	2.59	2.910(5)	100.0
C74	H74A	S21	0.99	2.83	3.804(4)	170.0

' = 1-x,1-y,1-z; '' = 1-x,2-y,1-z

Table S5: Drug susceptibility profile of the bacterial strains used in this work.

**Tested Drug**	*E. coli*	*S. aureus**	*S. haemolyticus*	*P. aeruginosa -01*	*P. aeruginosa-02*
Amikacin	**R**	-	-	S	S
Amoxicillin/ac. Clavulanic	**R**	-	-	-	-
Benzylpenicillin	-	**R**	-	-	-
Cefepime	-	-	-	S	S
Cefotaxime	S	-	-	-	-
Ceftaroline	-	S	-	-	-
Ceftazidime	S	-	-	S	S
Ciprofloxacin	-	-	-	S	S
Clindamycin	-	S	S	-	-
Colistin	-	-	-	S	S
Daptomycin	-	S	-	-	-
Ertapenem	S	-	-	**R**	**R**
Erythromycin	-	**R**	S	-	-
Fosfomycin	-	-	-	**R**	S
Fusidic acid	-	S	S	-	-
Gentamicin	-	S	S	S	S
Imipenem	-	-	-	S	S
Levofloxacin	-	S	S	-	-
Linezolid	-	S	S	-	-
Meropenem	S	-	-	S	S
Oxacillin	-	S	**R**	-	-
Piperacillin-tazobactam	S	-	-	S	S
Rifampicin	-	S	S	-	-
Teicoplanin	-	S	-	-	-
Tetracycline	-	S	-	-	-
Tigecycline	-	S	S	-	-
Trimethoprim-sulfamethoxazole	-	S	**R**	**R**	**R**
Vancomycin	-	S	S	-	-

Legend: drug susceptibility profile in according with AES EUCAST_Jannuary_2017 MIC values, (R = resistant, S= Sensitive) * = Methicillin resistant (MSRA) Strain. Antibiotic susceptibility was determined by Vitek-2 Compact system.

Table S6: MIC, MBC, and MBIC values (µg/mL) towards bacterial strains determined for $NiCl_2{\cdot}6H_2O$.

	MIC	MBC	MBIC
S. aureus	>119	>119	>119
S. haemoliticus	>119	>119	>119
E. coli	119	>119	0.119
P. aeruginosa	119	>119	0.119
C. albicans	119	>119	0.119
C. glabrata	59.5	>119	0.0595
C. kruseii	119	>119	0.119

Table S7: MIC, MBC, and MBIC values (mol/L) towards bacterial strains determined for $NiCl_2{\cdot}6H_2O$.

	MIC	MBC	MBIC
S. aureus	$>5{\cdot}10^{-4}$	$>5{\cdot}10^{-4}$	$>5{\cdot}10^{-4}$
S. haemoliticus	$>5{\cdot}10^{-4}$	$>5{\cdot}10^{-4}$	$>5{\cdot}10^{-4}$
E. coli	$5{\cdot}10^{-4}$	$>5{\cdot}10^{-4}$	$5{\cdot}10^{-4}$
P. aeruginosa	$5{\cdot}10^{-4}$	$>5{\cdot}10^{-4}$	$5{\cdot}10^{-4}$
C. albicans	$5{\cdot}10^{-4}$	$>5{\cdot}10^{-4}$	$5{\cdot}10^{-4}$
C. glabrata	$2.5{\cdot}10^{-4}$	$>2.5{\cdot}10^{-4}$	$2.5{\cdot}10^{-4}$
C. kruseii	$5{\cdot}10^{-4}$	$>5{\cdot}10^{-4}$	$5{\cdot}10^{-4}$

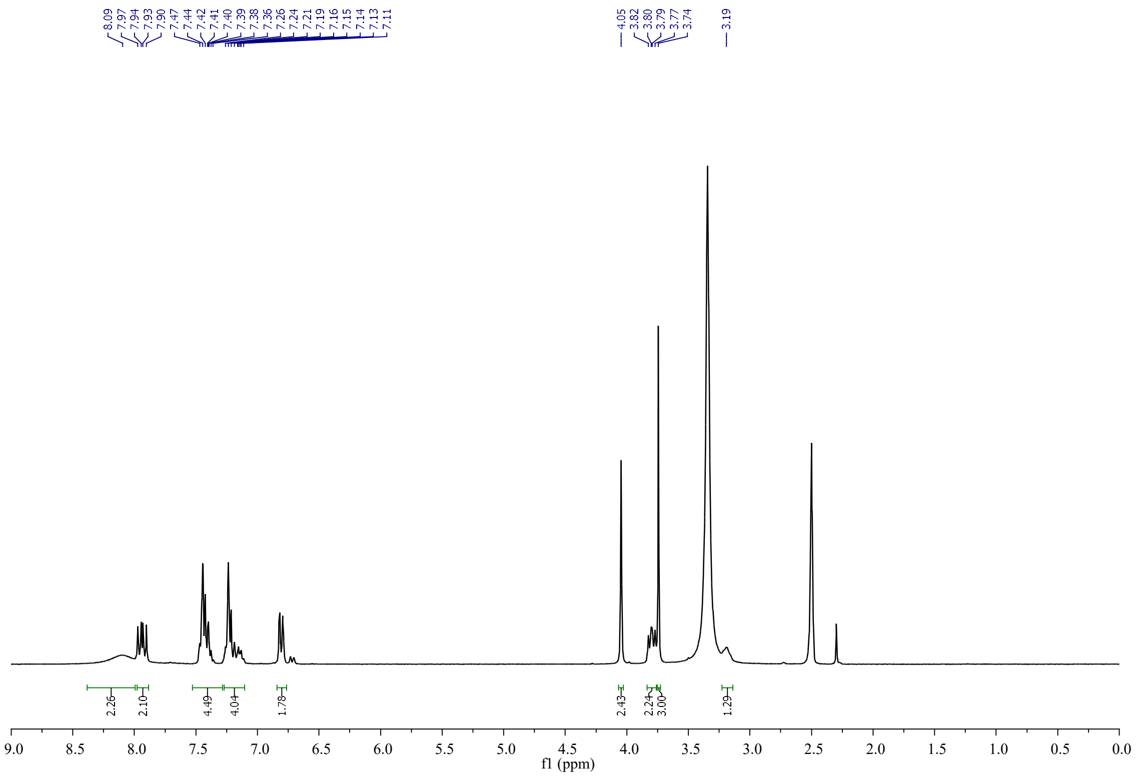

Figure S1: ^{1}H NMR spectrum of $(BzNH_3)$(BzNH-adtp) in DMSO-d_6 at 25 °C.

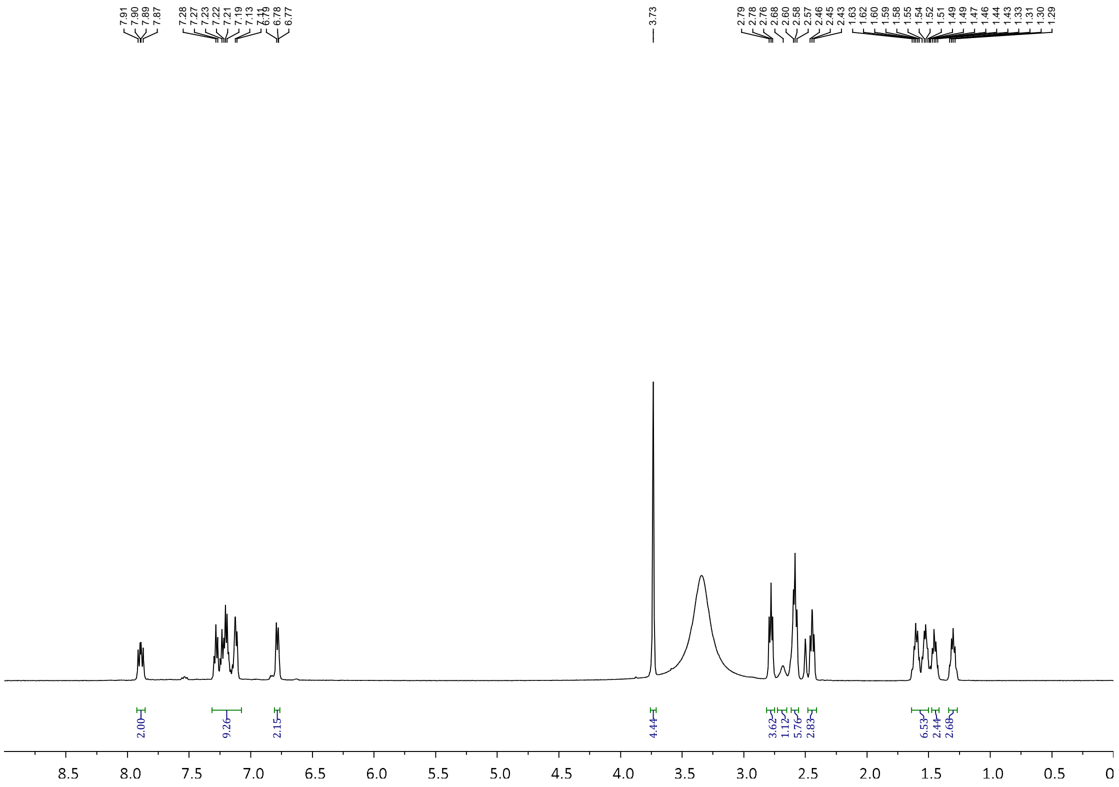

Figure S2: ^{1}H NMR spectrum of $(PhBuNH_3)$(PhBuNH-adtp) in DMSO-d_6 at 25 °C.

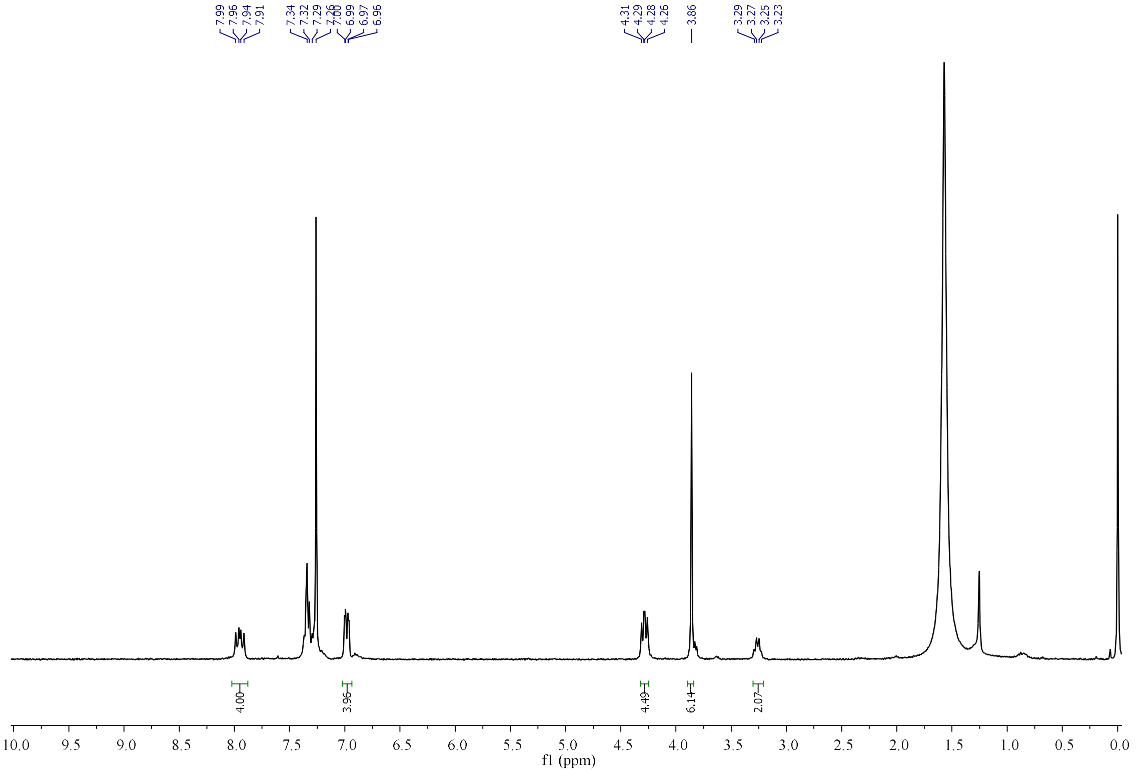

Figure S3: ^{1}H NMR spectrum of [Ni(BzNH-adtp)$_2$] in $CDCl_3$ at 25 °C.

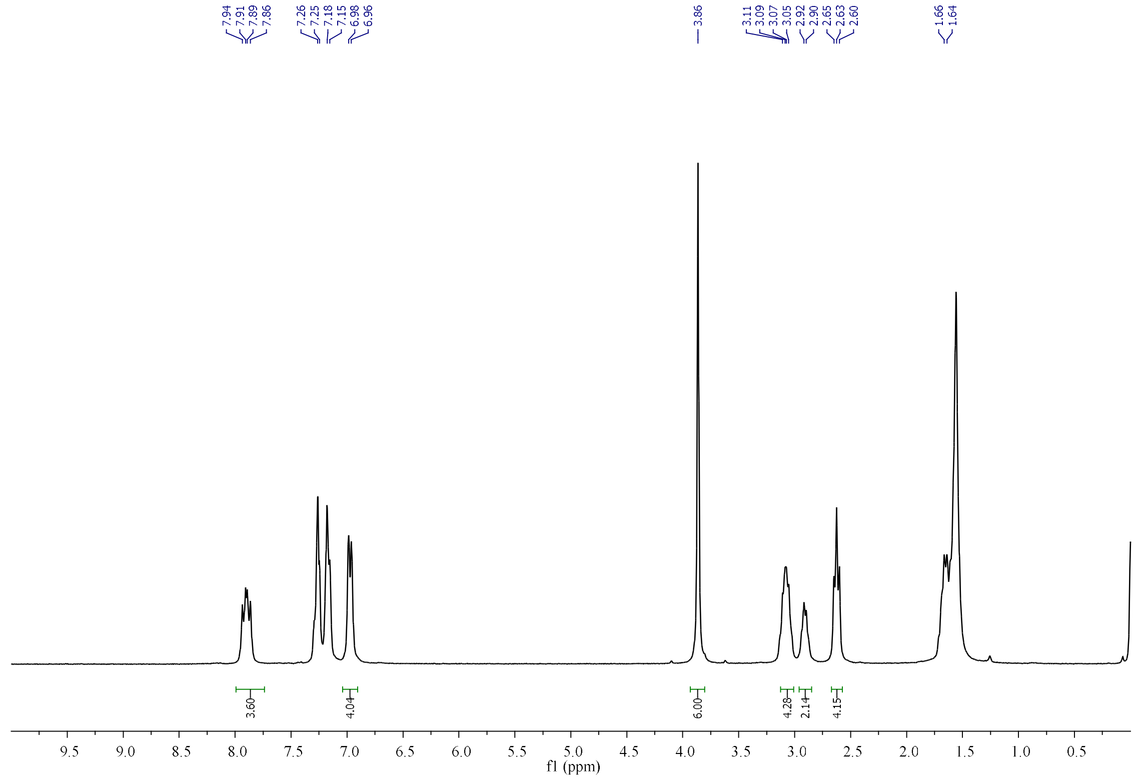

Figure S4: ^{1}H NMR spectrum of [Ni(PhBuNH-adtp)$_2$] in $CDCl_3$ at 25 °C.

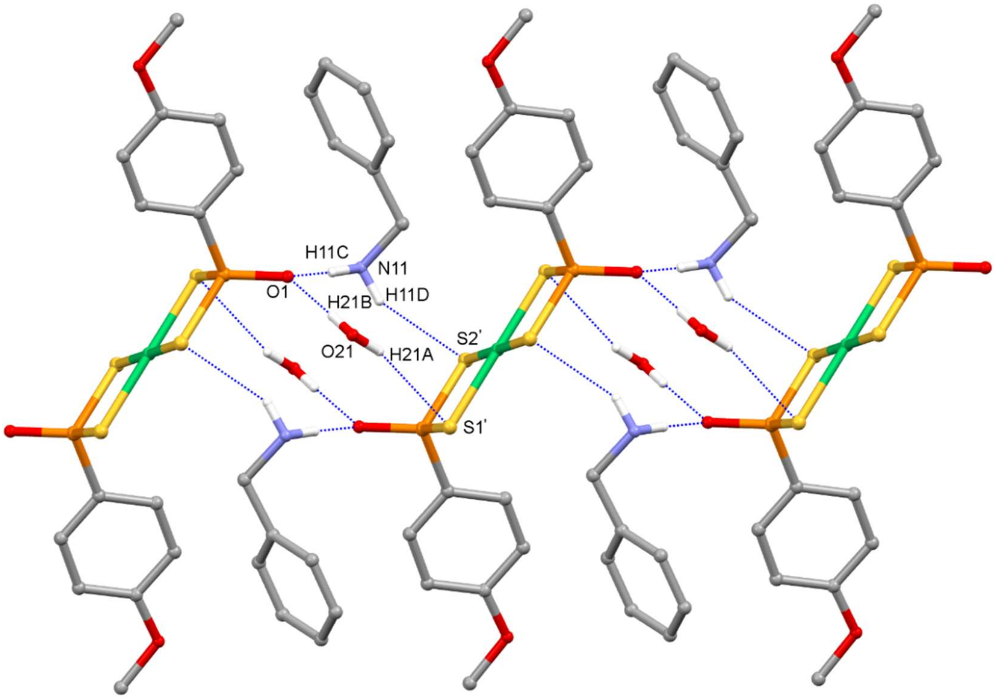

Figure S5: Packing view for $(BzNH_3)_2[Ni(dtp)_2]\cdot 2H_2O$ along the 010 direction.

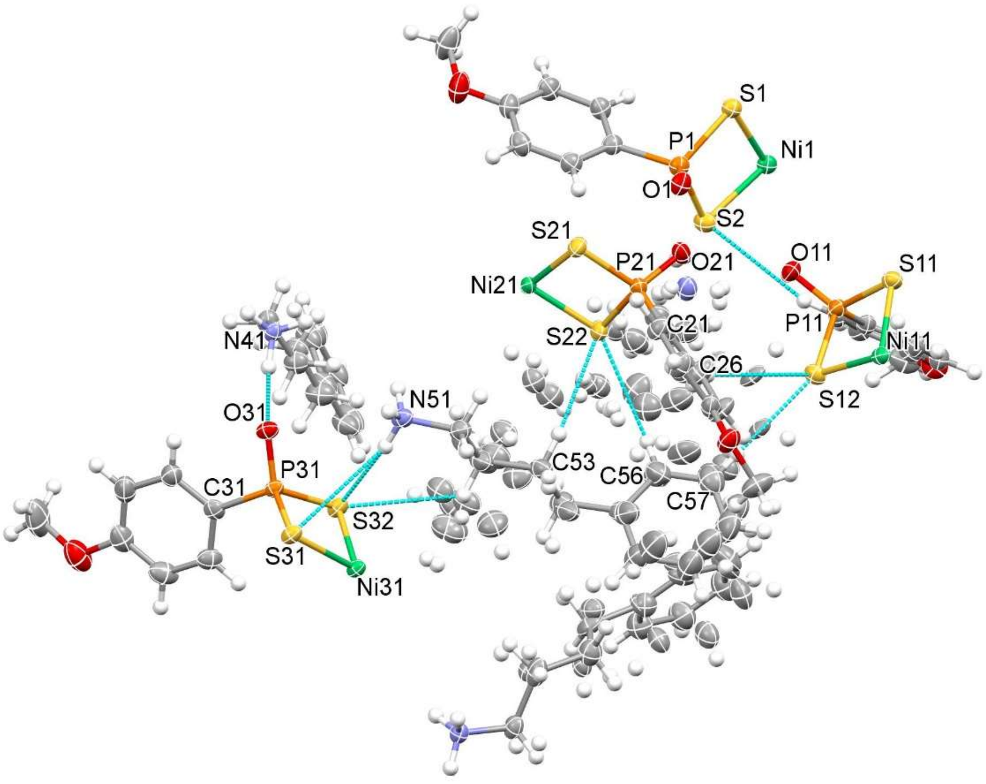

Figure S6: Contents and numbering scheme of the asymmetric unit of $(PhBuNH_3)_2[Ni(dtp)_2]\cdot 2H_2O$ thermal ellipsoids drawn at the 50% probability level, disorder shown.

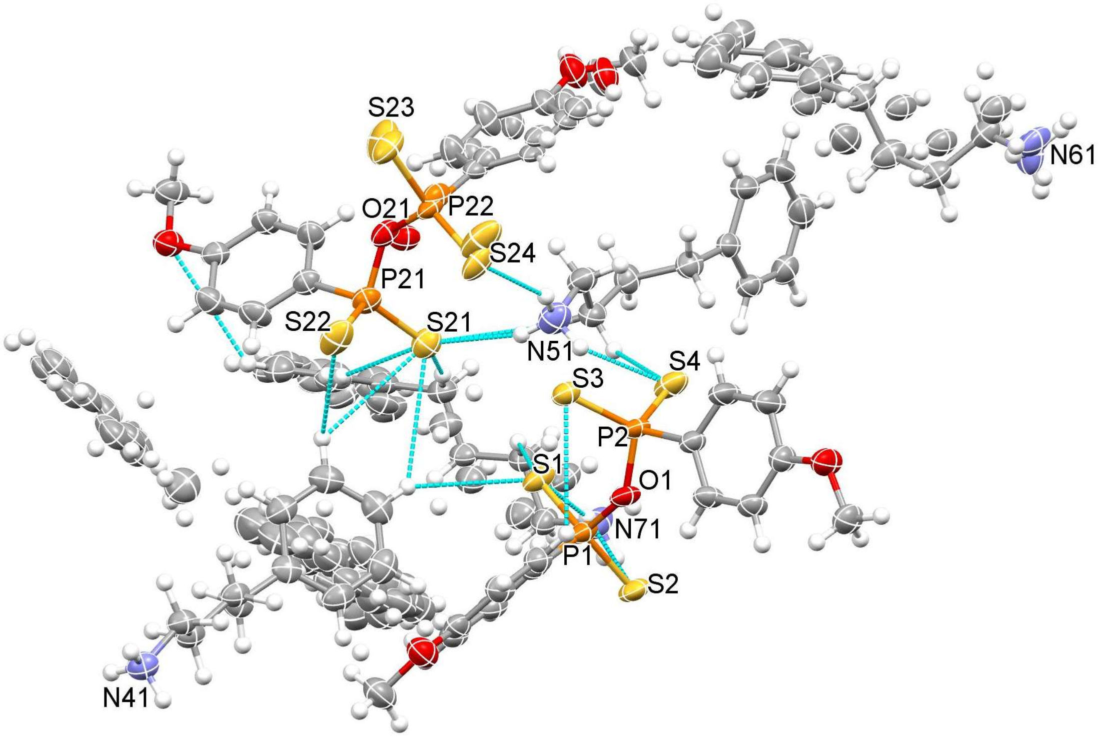

Figure S7: Contents and numbering scheme of the asymmetric unit of $(PhBuNH_3)_2[(ArPS_2)_2O]$ thermal ellipsoids drawn at the 50% probability level, disorder shown.

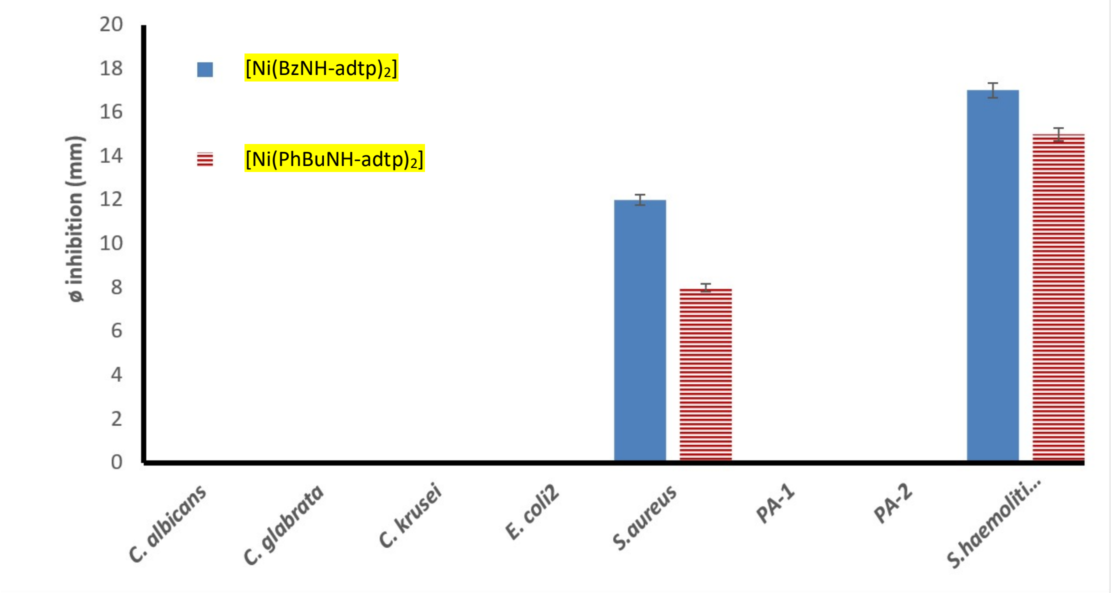

Figure S8: Antimicrobial activity, as inhibition diameter ø of $[Ni(BzNH\text{-}adtp)_2]$ and $[Ni(PhBuNH\text{-}adtp)_2]$ against eight different microbial species, C = [100 μg/mL]. The bars represent the mean and lines indicate the standard error of the mean.

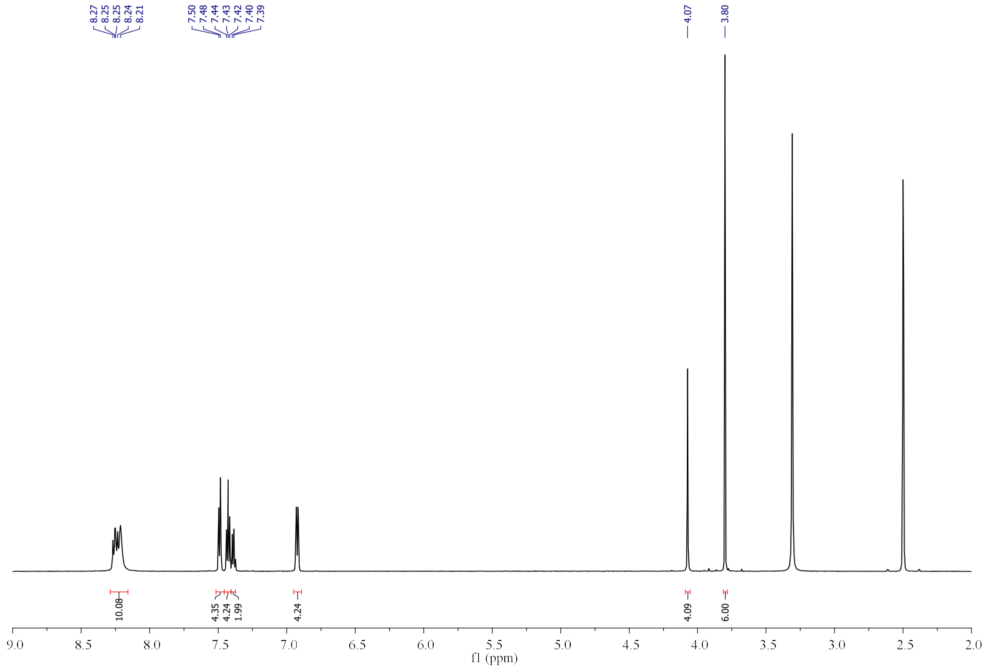

Figure S9: ^{1}H NMR spectrum of $(BzNH_3)_2[Ni(dtp)_2]$ in DMSO-d_6 at 25 °C.

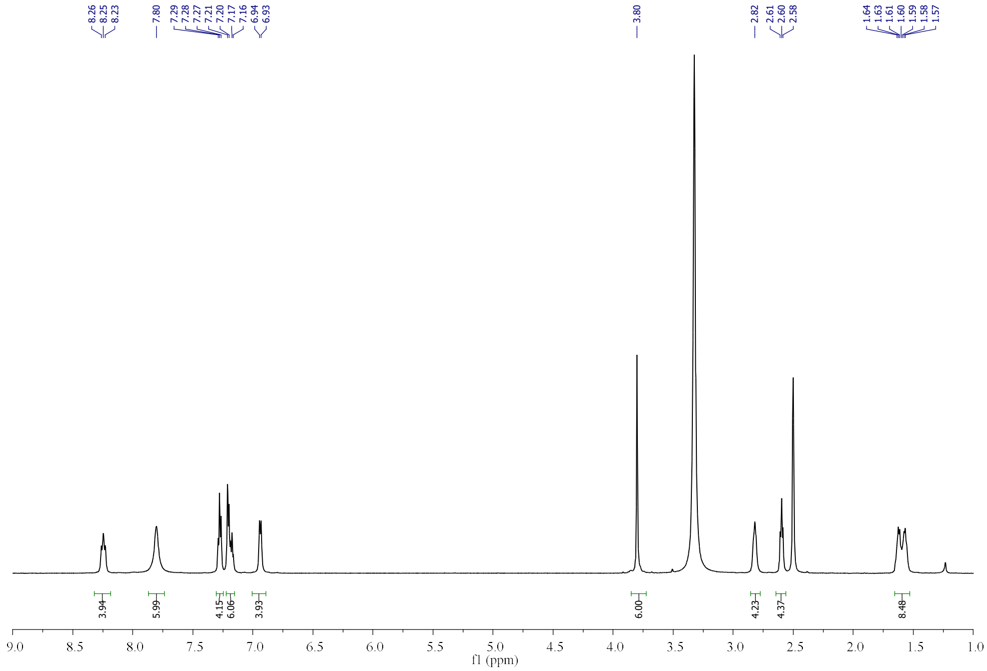

Figure S10: ^{1}H NMR spectrum of $(PhBuNH_3)_2[Ni(dtp)_2]$ in DMSO-d_6 at 25 °C.

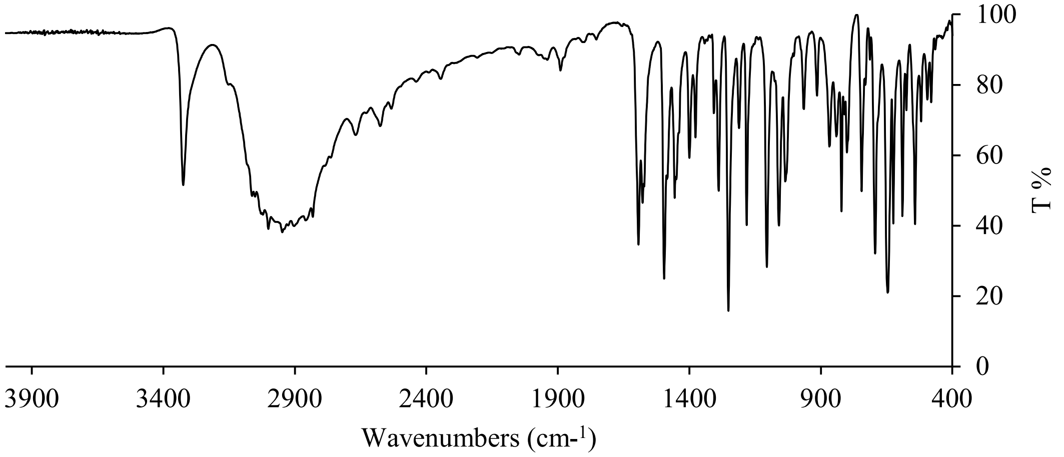

Figure S11: FT-IR spectrum of $(BzNH_3)$(BzNH-adtp).

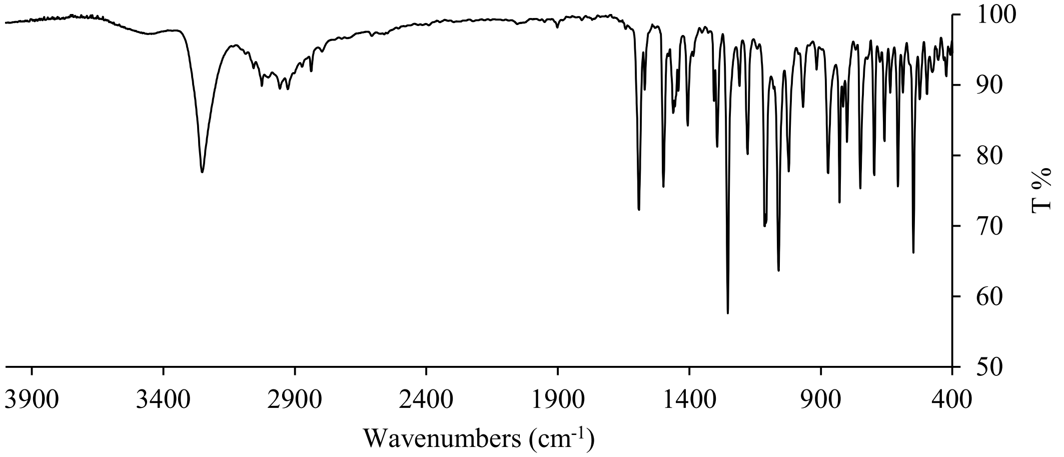

Figure S12: FT-IR spectrum of $[Ni(BzNH\text{-}adtp)_2]$.

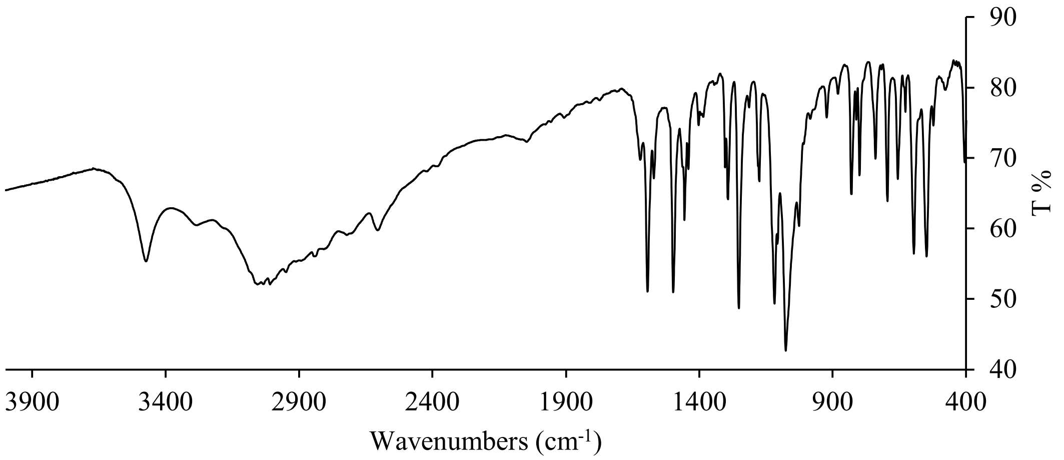

Figure S13: FT-IR spectrum of $(BzNH_3)_2[Ni(dtp)_2]$.

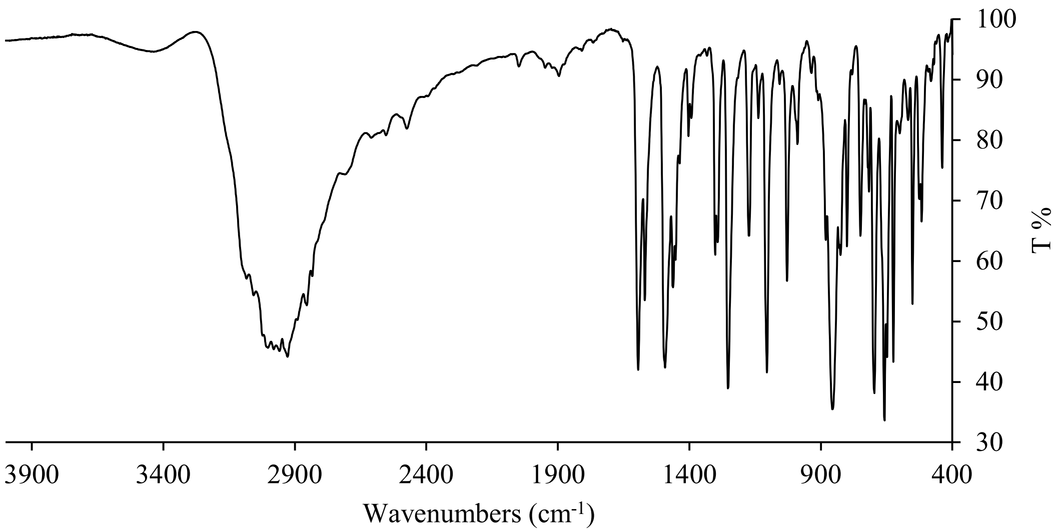

Figure S14: FT-IR spectrum of $(PhBuNH_3)$(PhBuNH-adtp).

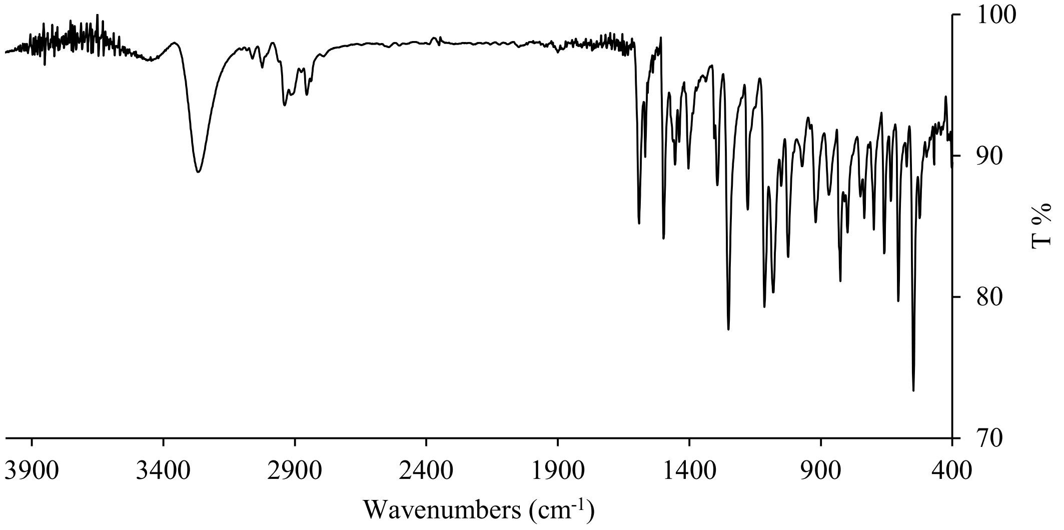

Figure S15: FT-IR spectrum of [Ni(PhBuNH-adtp)$_2$].

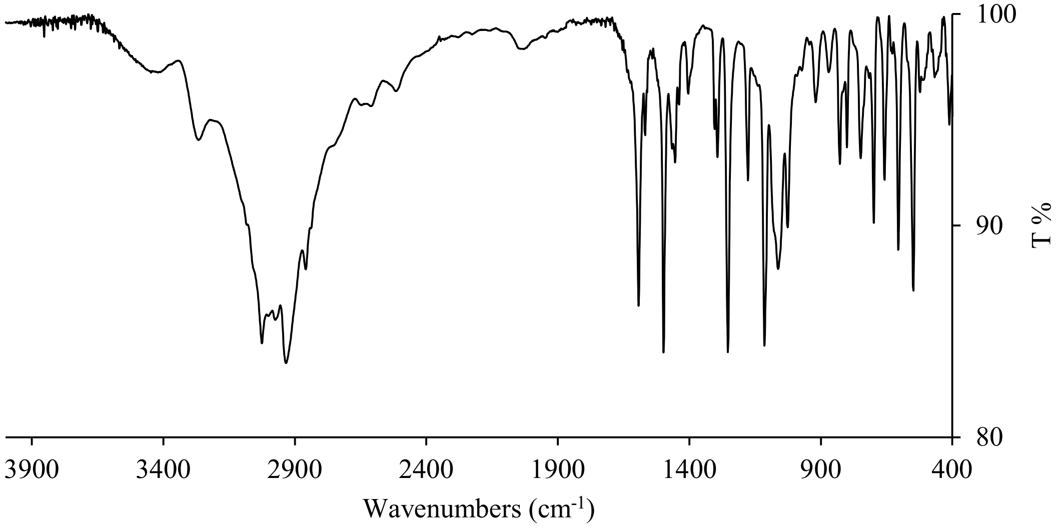

Figure S16: FT-IR spectrum of (PhBuNH$_3$)$_2$[Ni(dtp)$_2$].

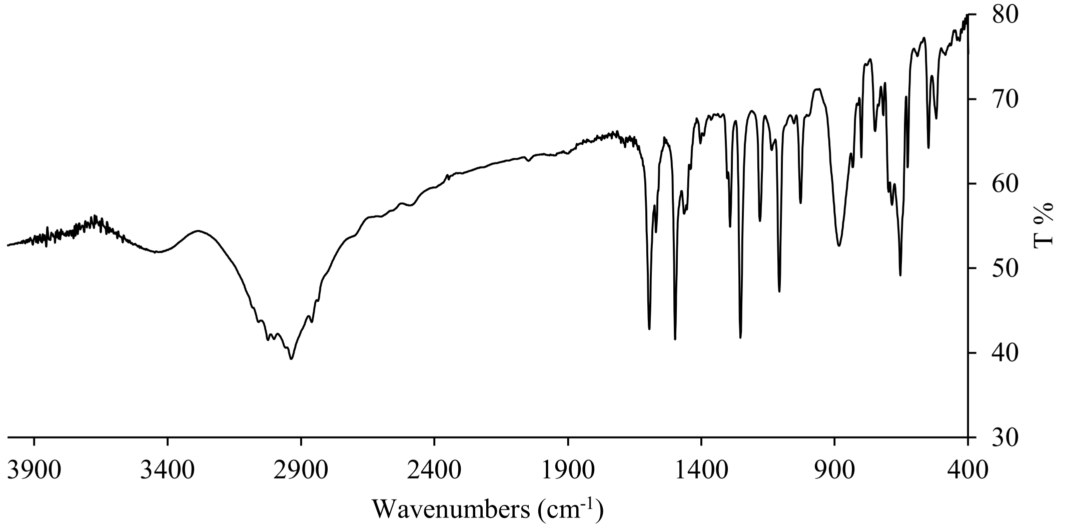

Figure S17: FT-IR spectrum of $(PhBuNH_3)_2[(ArPS_2)_2O]$.

checkCIF/PLATON report for $(PhBuNH_3)_2[(ArPS_2)_2O]$.

THIS REPORT IS FOR GUIDANCE ONLY. IF USED AS PART OF A REVIEW PROCEDURE FOR PUBLICATION, IT SHOULD NOT REPLACE THE EXPERTISE OF AN EXPERIENCED CRYSTALLOGRAPHIC REFEREE.

No syntax errors found. CIF dictionary Interpreting this report

Datablock: 2018ncs0299_r1_100k

Bond precision: C-C = 0.0043 A Wavelength=0.71075

Cell:
Temperature:
Volume
Space group
Hall group
Moiety formula

a=14.0833(3) b=15.6656(3) c=20.0356(4) alpha=96.779(2) beta=100.906(2) gamma=106.974(2) 100 K

Mu (mm-1) 0.350
F000 1678.0 F000’ 1681.31 h,k,lmax 18,20,26 Nref 18708 Tmin,Tmax 0.944,0.983 Tmin’ 0.914

0.350 1678.0

18,20,26
18643
0.448,1.000
Calculated
4080.13(16)
P -1
-P 1
Reported
4080.13(15)
P -1
-P 1

4(C14 H14 O3 P2 S4), 6(C102(C14 H14 O3 P2 S4), 3(C10

H16 N), 2(C5.74 H9.38
N0.60), 2(C4.2
C157 H208 N8 O12 P8 S16
3160.03

1.286

H16 N), C4.26 H6.62 N0.4,
C5.74 H9.3
C78.50 H104 N4 O6 P4 S8
1580.01

1.286

Sum formula
Mr
Dx,g cm-3 Z12

Correction method= # Reported T Limits: Tmin=0.448 Tmax=1.000
AbsCorr = GAUSSIAN

Data completeness= 0.997 Theta(max)= 27.485 R(reflections)= 0.0583(13259) wR2(reflections)= 0.1540(18643) S = 1.023 Npar= 1369

The following ALERTS were generated. Each ALERT has the format **test-name_ALERT_alert-type_alert-level**.

Click on the hyperlinks for more details of the test.

Alert level C

PLAT230_ALERT_2_C Hirshfeld Test Diff for
PLAT230_ALERT_2_C Hirshfeld Test Diff for
PLAT230_ALERT_2_C Hirshfeld Test Diff for
PLAT250_ALERT_2_C Large U3/U1 Ratio for Average U(i,j) Tensor ....
PLAT250_ALERT_2_C Large U3/U1 Ratio for Average U(i,j) Tensor ....
PLAT250_ALERT_2_C Large U3/U1 Ratio for Average U(i,j) Tensor ....
PLAT250_ALERT_2_C Large U3/U1 Ratio for Average U(i,j) Tensor ....
PLAT250_ALERT_2_C Large U3/U1 Ratio for Average U(i,j) Tensor ....
PLAT250_ALERT_2_C Large U3/U1 Ratio for Average U(i,j) Tensor ....
PLAT340_ALERT_3_C Low Bond Precision on C-C Bonds ....
PLAT411_ALERT_2_C Short Inter H...H Contact H53A ..H78A .
1-x,2-y,1-z =

Alert level G

FORMU01_ALERT_1_G There is a discrepancy between the atom counts in the
_chemical_formula_sum and _chemical_formula_moiety. This is
usually due to the moiety formula being in the wrong format.
Atom count from _chemical_formula_sum: C78.5 H104 N4 O6 P4 S8
Atom count from _chemical_formula_moiety:C68 H92 N4 O6 P4 S8

S22 --P21 .
S1 --P1 .
S4 --P2 .
6.7 s.u.
6.4 s.u.
6.3 s.u.
2.2 Note
2.2 Note
2.1 Note
2.1 Note
2.5 Note
2.4 Note
0.00426 Ang.
2.01 Ang.

2_676 Check

PLAT002_ALERT_2_G Number of Distance or Angle Restraints on AtSite
PLAT003_ALERT_2_G Number of Uiso or Uij Restrained non-H Atoms ...
PLAT007_ALERT_5_G Number of Unrefined Donor-H Atoms ....
PLAT042_ALERT_1_G Calc. and Reported MoietyFormula Strings Differ
PLAT045_ALERT_1_G Calculated and Reported Z Differ by a Factor ...
PLAT154_ALERT_1_G The s.u.'s on the Cell Angles are Equal ..(Note)

PLAT172_ALERT_4_G The CIF-Embedded .res File Contains DFIX Records
PLAT176_ALERT_4_G The CIF-Embedded .res File Contains SADI Records
PLAT178_ALERT_4_G The CIF-Embedded .res File Contains SIMU Records
PLAT187_ALERT_4_G The CIF-Embedded .res File Contains RIGU Records
PLAT301_ALERT_3_G Main Residue Disorder ....(Resd 1)
PLAT302_ALERT_4_G Anion/Solvent/Minor-Residue Disorder (Resd 3)
PLAT302_ALERT_4_G Anion/Solvent/Minor-Residue Disorder (Resd 6)
PLAT302_ALERT_4_G Anion/Solvent/Minor-Residue Disorder (Resd 7)
PLAT302_ALERT_4_G Anion/Solvent/Minor-Residue Disorder (Resd 8)
PLAT302_ALERT_4_G Anion/Solvent/Minor-Residue Disorder (Resd 9)
PLAT302_ALERT_4_G Anion/Solvent/Minor-Residue Disorder (Resd 10)
PLAT302_ALERT_4_G Anion/Solvent/Minor-Residue Disorder (Resd 11)
PLAT302_ALERT_4_G Anion/Solvent/Minor-Residue Disorder (Resd 12)
PLAT302_ALERT_4_G Anion/Solvent/Minor-Residue Disorder (Resd 13)
PLAT304_ALERT_4_G Non-Integer Number of Atoms in ...... Resd 6
PLAT304_ALERT_4_G Non-Integer Number of Atoms in ...... Resd 7
PLAT304_ALERT_4_G Non-Integer Number of Atoms in ...... Resd 8
PLAT304_ALERT_4_G Non-Integer Number of Atoms in ...... Resd 9
PLAT304_ALERT_4_G Non-Integer Number of Atoms in ...... Resd 10
PLAT304_ALERT_4_G Non-Integer Number of Atoms in ...... Resd 11
PLAT304_ALERT_4_G Non-Integer Number of Atoms in ...... Resd 12
PLAT304_ALERT_4_G Non-Integer Number of Atoms in ...... Resd 13
12 Note
165 Report
18 Report
Please Check
0.50 Check
0.002 Degree
4 Report
1 Report
6 Report
3 Report

52% Note

91% Note
100% Note
100% Note
100% Note
100% Note
100% Note
100% Note
100% Note
100% Note
15.72 Check
11.28 Check
4.89 Check
4.36 Check
4.05 Check
1.70 Check
5.35 Check
2.14 Check
132.6 Degree
136.4 Degree
137.6 Degree
3.16 Ang.
2_566 Check
3 Note
90 Check
PLAT395_ALERT_2_G Deviating X-O-Y
PLAT395_ALERT_2_G Deviating X-O-Y

PLAT395_ALERT_2_G Deviating X-O-Y
PLAT432_ALERT_2_G Short Inter X...Y Contact S4 ..C34
-x,1-y,1-z =
PLAT720_ALERT_4_G Number of Unusual/Non-Standard Labels ....
PLAT789_ALERT_4_G Atoms with Negative _atom_site_disorder_group #
Angle From 120 for O21
Angle From 120 for O21A
Angle From 120 for O1
PLAT811_ALERT_5_G No ADDSYM Analysis: Too Many Excluded Atoms ....
PLAT860_ALERT_3_G Number of Least-Squares Restraints ....
PLAT933_ALERT_2_G Number of OMIT Records in Embedded .res File ...

0 **ALERT level A** = Most likely a serious problem - resolve or explain

! Info
2674 Note
10 Note

0 **ALERT level B** = A potentially serious problem, consider carefully
11 **ALERT level C** = Check. Ensure it is not caused by an omission or oversight 38 **ALERT level G** = General information/check it is not something unexpected

4 ALERT type 1 CIF construction/syntax error, inconsistent or missing data
17 ALERT type 2 Indicator that the structure model may be wrong or deficient
3 ALERT type 3 Indicator that the structure quality may be low
23 ALERT type 4 Improvement, methodology, query or suggestion
2 ALERT type 5 Informative message, check

It is advisable to attempt to resolve as many as possible of the alerts in all categories. Often the minor alerts point to easily fixed oversights, errors and omissions in your CIF or refinement strategy, so attention to these fine details can be worthwhile. In order to resolve some of the more serious problems it may be necessary to carry out additional measurements or structure refinements. However, the purpose of your study may justify the reported deviations and the more serious of these should normally be commented upon in the discussion or experimental section of a paper or in the "special_details" fields of the CIF. checkCIF was carefully designed to identify outliers and unusual parameters, but every test has its limitations and alerts that are not important in a particular case may appear. Conversely, the absence of alerts does not guarantee there are no aspects of the results needing attention. It is up to the individual to critically assess their own results and, if necessary, seek expert advice.

Publication of your CIF in IUCr journals

A basic structural check has been run on your CIF. These basic checks will be run on all CIFs submitted for publication in IUCr journals (*Acta Crystallographica*, *Journal of Applied Crystallography*, *Journal of Synchrotron Radiation*); however, if you intend to submit to *Acta Crystallographica Section C* or *E* or *IUCrData*, you should make sure that full publication checks are run on the final version of your CIF prior to submission.

Publication of your CIF in other journals

Please refer to the *Notes for Authors* of the relevant journal for any special instructions relating to CIF submission.

PLATON version of 03/05/2019; check.def file version of 29/04/2019

Datablock 2018ncs0299_r1_100k - ellipsoid plot

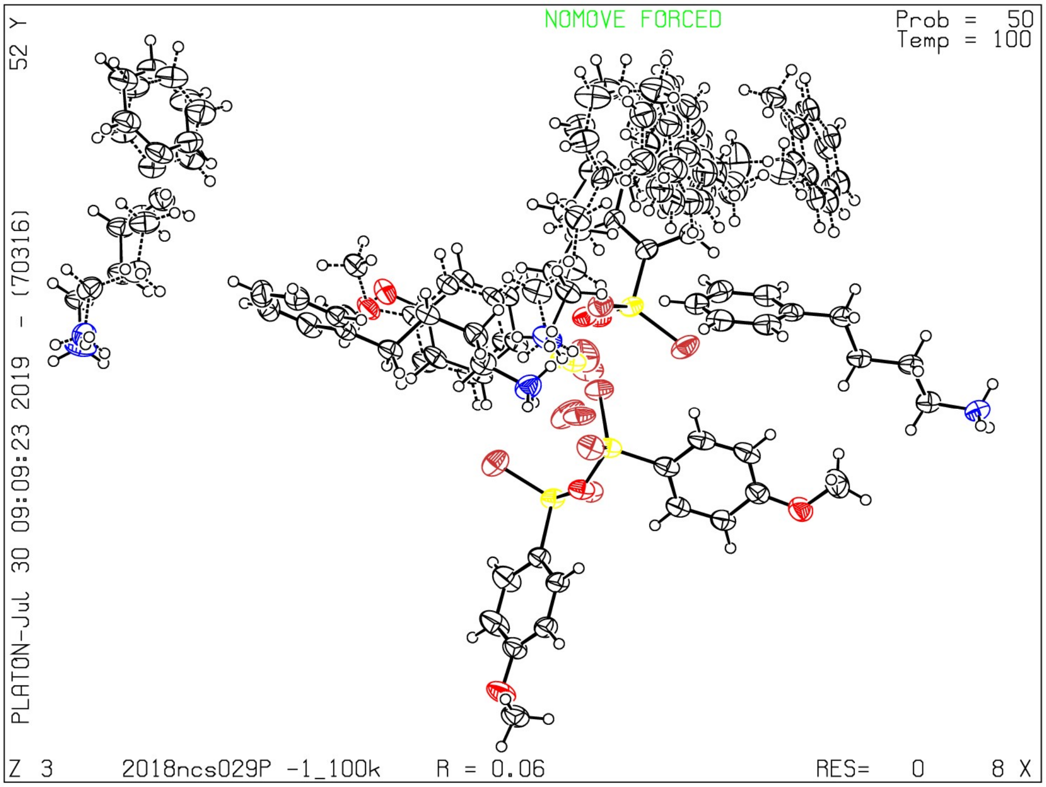
NOMOVE FORCED
Prob = 50
Temp = 100
52 Y
PLATON-JuL 30 09:09:23 2019 - (70316)
Z 3
2018ncs029P -1_100k
R = 0.06
RES= 0
8 X

checkCIF/PLATON report for $(BzNH_3)_2[Ni(dtp)_2]\cdot 2H_2O$.

THIS REPORT IS FOR GUIDANCE ONLY. IF USED AS PART OF A REVIEW PROCEDURE FOR PUBLICATION, IT SHOULD NOT REPLACE THE EXPERTISE OF AN EXPERIENCED CRYSTALLOGRAPHIC REFEREE.

No syntax errors found. CIF dictionary Interpreting this report

Datablock: 2018ncs0297_r1_100k

Bond precision: C-C = 0.0021 A Wavelength=0.71075

Cell:
Temperature:
Volume
Space group
Hall group
Moiety formula

a=6.8210(3) b=8.4029(2) c=15.5012(5) alpha=97.111(2) beta=90.729(3) gamma=102.606(3) 100 K

Mu (mm-1) 0.942
F000 390.0 F000' 391.16 h,k,lmax 8,10,20 Nref 3944 Tmin,Tmax 0.821,0.963 Tmin' 0.821

0.942 390.0

8,10,20
3941
0.500,1.000
Calculated
859.66(5)
P -1
-P 1
C14 H14 Ni O4 P2 S4, 2(C7
H10 N), 2(H2 O)
C28 H38 N2 Ni O6 P2 S4
747.47

1.444

Reported
859.66(5)
P -1
-P 1
C14 H14 Ni O4 P2 S4, 2(C7
H10 N), 2(H2 O)
C28 H38 N2 Ni O6 P2 S4
747.49

1.444

Sum formula
Mr
Dx,g cm-3 Z11

Correction method= # Reported T Limits: Tmin=0.500 Tmax=1.000
AbsCorr = GAUSSIAN

Data completeness= 0.999 Theta(max)= 27.486 R(reflections)= 0.0258(3591) wR2(reflections)= 0.0666(3941) S = 1.048 Npar= 216

The following ALERTS were generated. Each ALERT has the format **test-name_ALERT_alert-type_alert-level**.

Click on the hyperlinks for more details of the test.

Alert level C

PLAT094_ALERT_2_C Ratio of Maximum / Minimum Residual Density ....

Alert level G

PLAT007_ALERT_5_G Number of Unrefined Donor-H Atoms ....
PLAT171_ALERT_4_G The CIF-Embedded .res File Contains EADP Records

2.86 Report

4 Report

1 Report
8.0 s.u.
5.5 s.u.
5.5 s.u.
100% Note
100% Note
1.98 Check
1.02 Check

PLAT232_ALERT_2_G Hirshfeld Test Diff (M-X) Ni1
PLAT232_ALERT_2_G Hirshfeld Test Diff (M-X) Ni1
PLAT232_ALERT_2_G Hirshfeld Test Diff (M-X) Ni1
PLAT302_ALERT_4_G Anion/Solvent/Minor-Residue Disorder (Resd 3)
PLAT302_ALERT_4_G Anion/Solvent/Minor-Residue Disorder (Resd 4)
PLAT304_ALERT_4_G Non-Integer Number of Atoms in ...... Resd 3
PLAT304_ALERT_4_G Non-Integer Number of Atoms in ...... Resd 4

0 **ALERT level A** = Most likely a serious problem - resolve or explain
0 **ALERT level B** = A potentially serious problem, consider carefully
1 **ALERT level C** = Check. Ensure it is not caused by an omission or oversight 9 **ALERT level G** = General information/check it is not something unexpected

0 ALERT type 1 CIF construction/syntax error, inconsistent or missing data
4 ALERT type 2 Indicator that the structure model may be wrong or deficient
0 ALERT type 3 Indicator that the structure quality may be low
5 ALERT type 4 Improvement, methodology, query or suggestion
1 ALERT type 5 Informative message, check
--S1 .
--S2 .
--P1 .

It is advisable to attempt to resolve as many as possible of the alerts in all categories. Often the minor alerts point to easily fixed oversights, errors and omissions in your CIF or refinement strategy, so attention to these fine details can be

worthwhile. In order to resolve some of the more serious problems it may be necessary to carry out additional measurements or structure refinements. However, the purpose of your study may justify the reported deviations and the more serious of these should normally be commented upon in the discussion or experimental section of a paper or in the "special_details" fields of the CIF. checkCIF was carefully designed to identify outliers and unusual parameters, but every test has its limitations and alerts that are not important in a particular case may appear. Conversely, the absence of alerts does not guarantee there are no aspects of the results needing attention. It is up to the individual to critically assess their own results and, if necessary, seek expert advice.

Publication of your CIF in IUCr journals

A basic structural check has been run on your CIF. These basic checks will be run on all CIFs submitted for publication in IUCr journals (*Acta Crystallographica*, *Journal of Applied Crystallography*, *Journal of Synchrotron Radiation*); however, if you intend to submit to *Acta Crystallographica Section C* or *E* or *IUCrData*, you should make sure that full publication checks are run on the final version of your CIF prior to submission.

Publication of your CIF in other journals

Please refer to the *Notes for Authors* of the relevant journal for any special instructions relating to CIF submission.

PLATON version of 03/05/2019; check.def file version of 29/04/2019

Datablock 2018ncs0297_r1_100k - ellipsoid plot

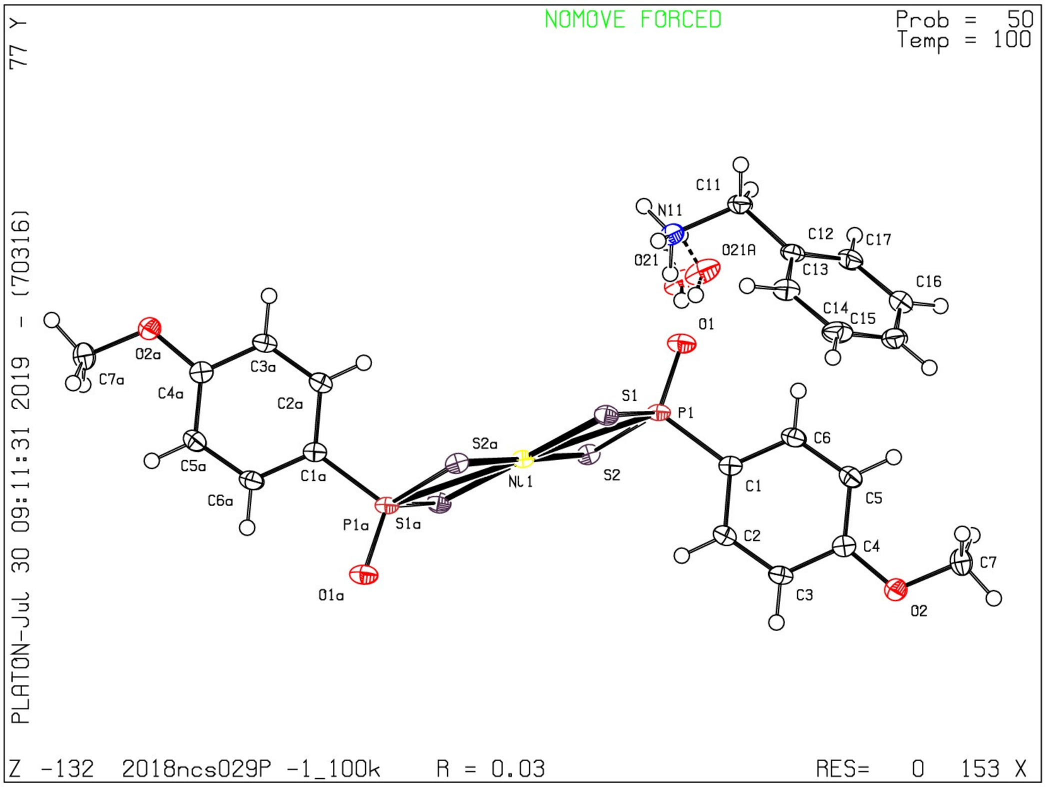

checkCIF/PLATON report for $(PhBuNH_3)_2[Ni(dtp)_2]$.

THIS REPORT IS FOR GUIDANCE ONLY. IF USED AS PART OF A REVIEW PROCEDURE FOR PUBLICATION, IT SHOULD NOT REPLACE THE EXPERTISE OF AN EXPERIENCED CRYSTALLOGRAPHIC REFEREE.

No syntax errors found. CIF dictionary Interpreting this report

Datablock: 2018ncs0294_r1_100k

```
Bond precision: C-C = 0.0041 A Wavelength=0.71075

Cell: a=15.6721(3) b=16.7478(4) c=18.1761(4) alpha=109.937(2)
beta=106.923(2) gamma=106.836(2)
```

```
Temperature:  100 K
                Calculated
Reported
3868.75(16)
P -1
-P 1
C14 H14 Ni O4 P2 S4, 2(C10
H16 N)
C34 H46 N2 Ni O4 P2 S4
795.62
1.366
Volume
Space group
Hall group
Moiety formula
3868.8(2)
P -1
-P 1
2(C10 H16 N), C14 H14 Ni
O4 P2 S4
Mu (mm-1)
F000
F000'
h,k,lmax
Nref
Tmin,Tmax
Tmin'
0.838
1672.0
1676.62
20,21,23
17759
0.914,0.934
0.881

0.838 1672.0

20,21,23
```

17089
0.630,1.000
C34 H46 N2 Ni O4 P2 S4
795.60
1.366

Sum formula
Mr
Dx,g cm-3 Z44

Correction method= # Reported T Limits: Tmin=0.630 Tmax=1.000
AbsCorr = GAUSSIAN

Data completeness= 0.962 Theta(max)= 27.485 R(reflections)= 0.0395(14095) wR2(reflections)= 0.1112(17089) S = 1.050 Npar= 1082

The following ALERTS were generated. Each ALERT has the format **test-name_ALERT_alert-type_alert-level**.

Click on the hyperlinks for more details of the test.

Alert level C

```
CRYSC01_ALERT_1_C The word below has not been recognised as a standard
            identifier.
            deep
PLAT094_ALERT_2_C Ratio of Maximum / Minimum Residual Density ....
PLAT234_ALERT_4_C Large Hirshfeld Difference C61      --C62A     .
PLAT411_ALERT_2_C Short Inter H...H Contact  H52A     ..H76B     .
```

x,y,z =

Alert level G

```
PLAT002_ALERT_2_G Number of Distance or Angle Restraints on AtSite
PLAT003_ALERT_2_G Number of Uiso or Uij Restrained non-H Atoms ...
PLAT007_ALERT_5_G Number of Unrefined Donor-H Atoms ..............
PLAT042_ALERT_1_G Calc. and Reported MoietyFormula Strings  Differ
PLAT152_ALERT_1_G The Supplied and Calc. Volume s.u. Differ by ...
PLAT154_ALERT_1_G The s.u.'s on the Cell Angles are Equal ..(Note)
PLAT171_ALERT_4_G The CIF-Embedded .res File Contains EADP Records
PLAT176_ALERT_4_G The CIF-Embedded .res File Contains SADI Records
PLAT178_ALERT_4_G The CIF-Embedded .res File Contains SIMU Records
PLAT187_ALERT_4_G The CIF-Embedded .res File Contains RIGU Records

PLAT232_ALERT_2_G Hirshfeld Test Diff (M-X)  Ni1
PLAT232_ALERT_2_G Hirshfeld Test Diff (M-X)  Ni21
PLAT232_ALERT_2_G Hirshfeld Test Diff (M-X)  Ni21
PLAT232_ALERT_2_G Hirshfeld Test Diff (M-X)  Ni31
PLAT301_ALERT_3_G Main Residue  Disorder ..............(Resd  1  )
PLAT301_ALERT_3_G Main Residue  Disorder ..............(Resd  6  )
PLAT301_ALERT_3_G Main Residue  Disorder ..............(Resd  7  )
PLAT432_ALERT_2_G Short Inter X...Y Contact  C37      ..C66A
                                                    x,y,-1+z  =
PLAT811_ALERT_5_G No ADDSYM Analysis: Too Many Excluded Atoms ....
PLAT860_ALERT_3_G Number of Least-Squares Restraints .............
PLAT933_ALERT_2_G Number of OMIT Records in Embedded .res File ...
     2.06 Report
     0.16 Ang.
     2.12 Ang.
```

```
1_555 Check

       34 Note
       64 Report
       18 Report
   Please Check
        4 Units
    0.002 Degree
        3 Report
        2 Report
        2 Report
        2 Report
      5.5 s.u.
      5.5 s.u.
      5.5 s.u.
      6.7 s.u.
      91% Note
      82% Note
      64% Note
     3.19 Ang.
1_554 Check
        ! Info
     1299 Note
        5 Note
```

0 **ALERT level A** = Most likely a serious problem - resolve or explain
0 **ALERT level B** = A potentially serious problem, consider carefully
4 **ALERT level C** = Check. Ensure it is not caused by an omission or oversight

21 **ALERT level G** = General information/check it is not something unexpected

```
 4 ALERT type 1 CIF construction/syntax error, inconsistent or missing data
10 ALERT type 2 Indicator that the structure model may be wrong or deficient
 4 ALERT type 3 Indicator that the structure quality may be low
 5 ALERT type 4 Improvement, methodology, query or suggestion
 2 ALERT type 5 Informative message, check
--S2       .
--S21      .
--S22      .
--S31      .
```

It is advisable to attempt to resolve as many as possible of the alerts in all categories. Often the minor alerts point to easily fixed oversights, errors and omissions in your CIF or refinement strategy, so attention to these fine details can be worthwhile. In order to resolve some of the more serious problems it may be necessary to carry out additional measurements or structure refinements. However, the purpose of your study may justify the reported deviations and the more serious of these should normally be commented upon in the discussion or experimental section of a paper or in the "special_details" fields of the CIF. checkCIF was carefully designed to identify outliers and unusual parameters, but every test has its limitations and alerts that are not important in a particular case may appear. Conversely, the absence of alerts does not guarantee there are no aspects of the results needing attention. It is up to the individual to critically assess their own results and, if necessary, seek expert advice.

Publication of your CIF in IUCr journals

A basic structural check has been run on your CIF. These basic checks will be run on all CIFs submitted for publication in IUCr journals (*Acta Crystallographica*, *Journal of Applied Crystallography*, *Journal of Synchrotron Radiation*); however, if you intend to submit to *Acta Crystallographica Section C* or *E* or *IUCrData*, you should make sure that full publication checks are run on the final version of your CIF prior to submission.

Publication of your CIF in other journals

Please refer to the *Notes for Authors* of the relevant journal for any special instructions relating to CIF submission.

PLATON version of 03/05/2019; check.def file version of 29/04/2019

Datablock 2018ncs0294_r1_100k - ellipsoid plot

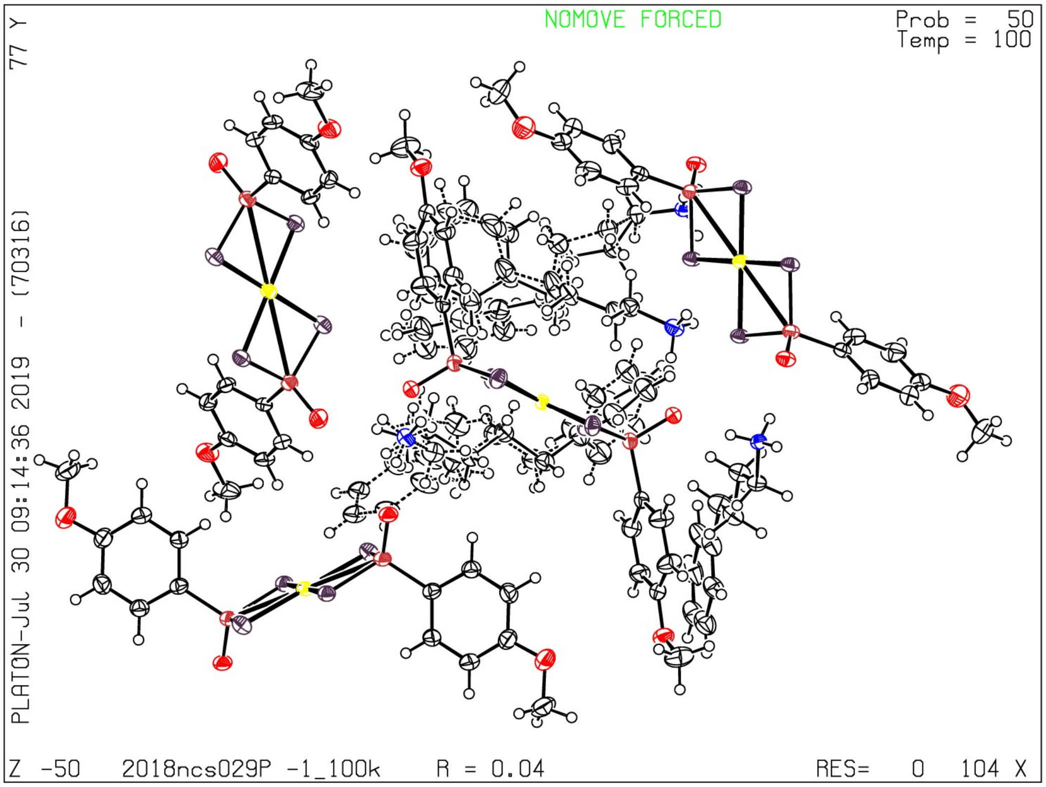

Supplement: Supplementary file 1 [file molecules-25-02052-s001.pdf]
